# Adaptive Complex Signal Average Diffusion-Weighted MR Imaging of the Liver: Utility in Breath-Hold Imaging: A Retrospective Single-Center Study

**DOI:** 10.3390/tomography12060084

**Published:** 2026-06-09

**Authors:** Masahiro Tanabe, Haruki Furutani, Miwa Matsukuma, Mayumi Higashi, Yuto Takemura, Jo Ishii, Masatoshi Yamane, Katsuyoshi Ito

**Affiliations:** 1 Department of Radiology, Yamaguchi University Graduate School of Medicine, 1-1-1 Minami-Kogushi, Ube 755-8505, Japan; 2 Department of Radiological Technology, Yamaguchi University Hospital, Ube 755-8505, Japan

**Keywords:** diffusion-weighted imaging, adaptive complex signal averaging, breath-hold, liver, apparent diffusion coefficient

## Abstract

**Objectives:** This study evaluated the utility of adaptive complex signal average (ACSA) diffusion-weighted imaging (DWI) specifically in breath-hold (BH) liver imaging, with a focus on signal intensity (SI) improvement, intrahepatic signal homogeneity, and apparent diffusion coefficient (ADC) behavior, and compared these findings with conventional non-ACSA DWI and free-breathing (FB) ACSA DWI. **Methods:** This retrospective study included 62 patients (mean age, 67.8 ± 13.6 years; 27 women) who underwent liver MRI with both FB and BH DWI on a 3-T system. Non-ACSA images were generated using conventional magnitude reconstruction, and ACSA images were reconstructed from identical raw data. SI, signal-to-noise ratio (SNR) and ADC were measured in the left lateral segment and right hepatic lobe. The signal intensity difference ratio (SIDR) between ACSA and non-ACSA, signal intensity ratio (SIR) and ADC ratio between right lobe and lateral segment were calculated. **Results:** In both FB and BH imaging, SI and SNR in both liver regions were significantly higher on ACSA DWI than on non-ACSA DWI (*p* < 0.01). ADC values were significantly lower with ACSA. SIDR was significantly higher in the left lateral segment (*p* < 0.01), indicating greater SI improvement in motion-prone regions. SIR and ADC ratios between lobes were significantly smaller with ACSA in both respiratory conditions (*p* < 0.01). FB-ACSA showed smaller SIR than BH-ACSA, while ADC ratios did not differ. **Conclusions:** ACSA DWI significantly improves SI, intrahepatic uniformity, and ADC reliability even under BH liver imaging. BH ACSA DWI may represent a potentially useful application complementary to FB ACSA DWI, supporting its consideration as a post-processing strategy for improving qualitative and quantitative liver DWI in future investigations.

## Simple Summary

This study explores a new imaging technique called adaptive complex signal average diffusion-weighted imaging (ACSA DWI) for liver MRI. Conventional methods often struggle with motion artifacts, especially in the left lateral segment of the liver, leading to unclear images and inaccurate measurements. ACSA DWI improves image clarity and consistency, even during breath-hold scans, by reducing noise and stabilizing signals in motion-prone regions. These advancements could enhance the detection of liver diseases and improve diagnostic accuracy in clinical practice.

## 1. Introduction

Diffusion-weighted imaging (DWI) is an essential component of liver MRI, providing a noninvasive assessment of tissue diffusivity and facilitating the detection and characterization of focal liver lesions [1,2]. However, conventional DWI is susceptible to respiratory motion and cardiac pulsation, particularly in the left lateral segment of the liver, resulting in signal loss, poor signal uniformity, and inaccurate apparent diffusion coefficient (ADC) measurements [3,4]. Breath-hold (BH) DWI has been introduced as a practical strategy to minimize respiratory motion and shorten examination time [5]. Nevertheless, BH acquisition inherently limits the number of excitations and remains vulnerable to residual motion, especially cardiac-induced motion, leading to persistent signal heterogeneity within the liver [3]. These limitations reduce the reliability of both qualitative image interpretation and quantitative diffusion analysis.

Adaptive complex signal average (ACSA) reconstruction is a post-processing technique that combines adaptive weighted averaging and complex-domain signal averaging. Adaptive averaging selectively down-weights motion-corrupted, low-intensity repetitions, whereas complex averaging suppresses the Rician noise floor inherent to magnitude-reconstructed DWI [6,7,8,9,10]. While previous studies have demonstrated the utility of ACSA DWI with free-breathing (FB) techniques in improving signal intensity (SI), signal-to-noise ratio (SNR), and ADC stability of the liver [7,8,9], its effectiveness in the constrained setting of BH liver DWI has not been fully clarified. The purpose of this study was therefore to evaluate the utility of ACSA DWI specifically in BH liver imaging, with a focus on SI improvement, intrahepatic signal homogeneity, and ADC behavior, and to compare these findings with conventional non-ACSA DWI and FB ACSA DWI.

## 2. Materials and Methods

### 2.1. Study Design and Population

**Figure 1:**
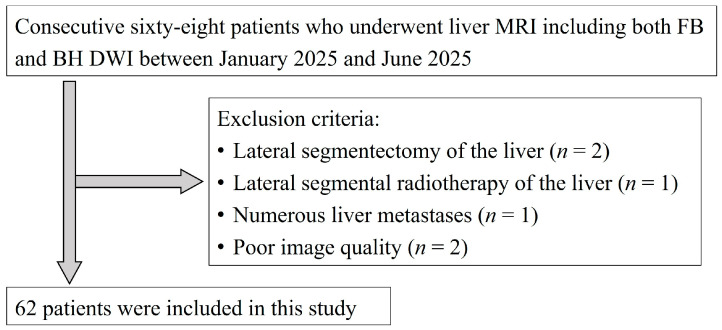
Patient selection flowchart.

This retrospective, single-center study was approved by the institutional review board, and the requirement for written informed consent was waived. Consecutive sixty-eight patients who underwent liver MRI including both FB and BH DWI between January 2025 and June 2025 were initially eligible for inclusion. For all patients, DWI datasets reconstructed using both ACSA and conventional non-ACSA methods were available for analysis. Patients were excluded according to the following criteria: history of lateral segmentectomy (*n* = 2) and lateral segmental radiotherapy (*n* = 1), numerous liver metastases (*n* = 1), or poor image quality (*n* = 2). After applying these exclusion criteria, a total of 62 patients (mean age, 67.8 ± 13.6 years; 35 men and 27 women) were included in this study Figure 1). Demographics of the study population are shown in Table 1.

### 2.2. MRI Acquisition

All MRI examinations were performed using a 3T MR system (Vantage Centurian; Canon Medical Systems, Otawara, Japan) with a phased-array body coil. BH DWI was acquired using a two-dimensional (2D), spin-echo (SE) echo-planar imaging (EPI) sequence with b-values of 0 and 1000 s/mm^2^. The imaging parameters were as follows; repetition time (TR)/echo time (TE), 1500/41 ms; flip angle, 90°; field of view (FOV), 300 × 380 mm; acquisition matrix, 480 × 606 using Precise IQ Engine (PIQE) algorithm [11]; bandwidth, 3125 Hz/pixel; slice thickness, 6 mm; number of slices, 24; parallel imaging factor, 2; number of excitation (NEX), 3. To cover the entire liver, four 20 s BH scans are performed, with a total acquisition time of 80 s. FB DWI was also obtained using a 2D-SE EPI sequence with b-values of 0 and 1000 s/mm^2^. The imaging parameters were as follows: TR/TE, 1663/51 ms; flip angle, 90°; FOV, 280 × 380 mm; matrix, 600 × 816 using PIQE algorithm; bandwidth, 3125 Hz/pixel; slice thickness, 5 mm; number of slices, 35; parallel imaging factor, 2; NEX, 2 (*n* = 10), 3 (*n* = 23), or 4 (*n* = 29); acquisition time, 85, 120, or 135 sec. The imaging parameters were not identical between BH and FB sequences because they were optimized separately for each acquisition strategy in routine clinical practice. Conventional magnitude-reconstructed DWI images (non-ACSA DWI) were first generated. Subsequently, ACSA DWI images were reconstructed from the same raw data using a deep learning-based algorithm incorporating adaptive averaging and complex-domain signal averaging.

### 2.3. Image Analysis

Quantitative image analysis was performed using a picture archiving and communication system (PACS; Shade Quest; FUJIFILM Medical Solutions, Tokyo, Japan) by two radiologists (H.F. and J.I.) with 2 years of experience in radiology. All measurements were subsequently reviewed and finalized by a board-certified radiologist (M.T.) with 24 years of experience in abdominal imaging. Both readers were blinded to MRI acquisition parameters and to all clinical information, including patient identifiers, medical history, final diagnoses, and findings from other imaging modalities. To assess intrahepatic homogeneity of SI and diffusion metrics, regions of interest (ROIs) were placed at four predefined sites in the liver: the peripheral and central portions of the left lateral segment, and the peripheral and central portions of the right hepatic lobe. ROIs were placed at identical anatomical locations on both DWI images and ADC maps for ACSA DWI and non-ACSA DWI using a copy-and-paste function. ROIs were also matched as closely as possible between FB and BH acquisitions on both DWI images and ADC maps. Using ROI measurements, SI, signal-to-noise ratio (SNR) and ADC values were obtained for each hepatic site. SNR was defined as the SI of the liver divided by the standard deviation (SD) of the spinal erector muscle. For both FB and BH imaging, SI and ADC values were compared between ACSA DWI and non-ACSA DWI. The signal intensity difference ratio (SIDR), defined as [(SI of ACSA − SI of non-ACSA)/SI of non-ACSA] × 100, was calculated and compared between the peripheral left lateral segment and the central right hepatic lobe for both FB-DWI and BH-DWI acquisitions. In addition, the signal intensity ratio (SIR), defined as (SI of liver, right lobe, center)/(SI of liver, lateral segment, periphery), and the ADC ratio, defined as (ADC of liver, right lobe, center)/(ADC of liver, lateral segment, periphery), were calculated to evaluate inter-lobar differences in SI and diffusion properties. The SIR was intentionally defined using the right lobe center (a region minimally affected by cardiac motion) and the lateral segment periphery (the region most susceptible to cardiac pulsation-related signal loss). This asymmetric pairing was chosen to maximize sensitivity to motion-induced signal degradation. These ratios were compared between ACSA DWI and non-ACSA DWI for both FB and BH imaging. Finally, the SIR and ADC ratio were also compared between FB-ACSA DWI and BH-ACSA DWI.

### 2.4. Statistical Analysis

Continuous variables are presented as median values (25th and 75th percentile), as appropriate. Normality was assessed using the Shapiro–Wilk test. Because most quantitative parameters did not follow a normal distribution, nonparametric analyses were applied. For both FB and BH acquisitions, paired comparisons of SI, SNR and ADC values between ACSA DWI and non-ACSA DWI were performed using the Wilcoxon signed-rank test. The SIDR and the inter-lobar ratios, including the SIR and ADC ratio, were also compared between ACSA and non-ACSA DWI using the Wilcoxon signed-rank test for each respiratory condition (FB and BH). In addition, the SIR and ADC ratio were compared between FB-ACSA DWI and BH-ACSA DWI using the Wilcoxon signed-rank test. Inter-reader reliability was assessed using the intraclass correlation coefficient (ICC), which was interpreted according to conventional thresholds (poor < 0.5, moderate 0.5–0.75, good 0.75–0.9, and excellent > 0.9). All statistical analyses were performed using JMP version 19 (JMP Statistical Discovery LLC, Cary, NC, USA) and SPSS version 31 (IBM Corp., Armonk, NY, USA). A two-sided *p* value < 0.05 was considered statistically significant.

## 3. Results

**Figure 2:**
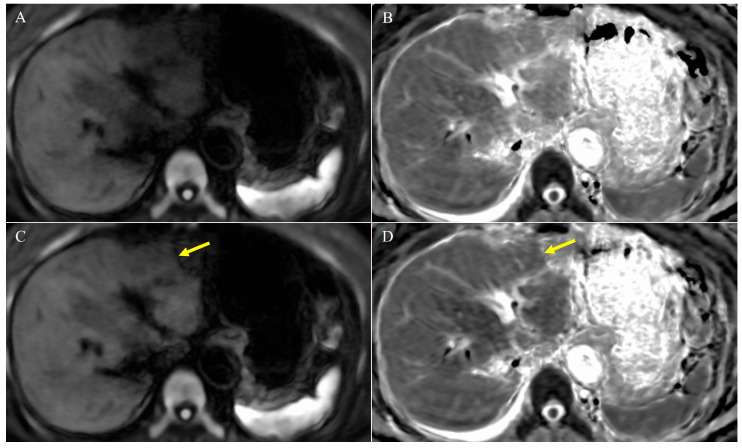
Comparison of BH non-ACSA (conventional) DWI (**A**) and corresponding ADC map (**B**) and BH-ACSA DWI (**C**) and corresponding ADC map (**D**). On BH-ACSA DWI, the signal intensity of the liver parenchyma in the left lateral segment showed a distinct signal increase (arrow in (**C**)) compared to BH non-ACSA DWI, with signal intensity comparable to that of the right lobe. In the BH-ACSA ADC map, the overestimation of ADC values observed in the lateral segment on the BH non-ACSA ADC map is improved, and the inter-lobar difference in ADC values is scarcely observed (arrow in (**D**)).

**Figure 3:**
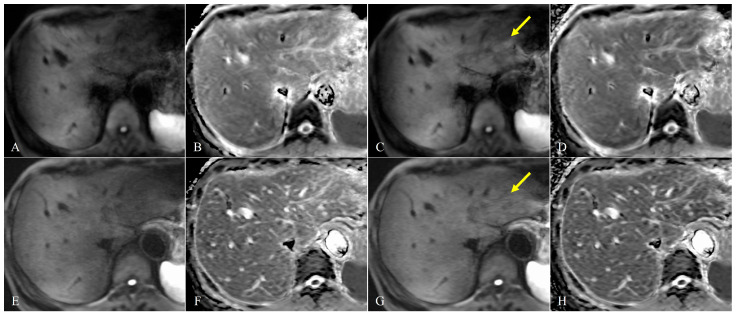
(**A**) BH non-ACSA (conventional) DWI and (**B**) ADC map, (**C**) BH-ACSA DWI and (**D**) ADC map, (**E**) FB non-ACSA (conventional) DWI and (**F**) ADC map, and (**G**) FB-ACSA DWI and (**H**) ADC map. In both FB and BH imaging, the signal intensity of the liver parenchyma in the left lateral segment on ACSA DWI showed a distinct signal increase (arrow in (**C**,**G**)) compared to non-ACSA DWI. Overestimated ADC values of the lateral segment on the non-ACSA ADC map are improved on the ACSA ADC map in both FB and BH imaging. In the comparison between BH-ACSA DWI and FB-ACSA DWI, the SIR between the right hepatic lobe and left lateral segment was smaller on FB-ACSA DWI (1.74) than on BH-ACSA DWI (3.28) (arrow in (**C**,**G**)).

In both FB and BH acquisitions, the SI and SNR of the left lateral hepatic segment and the right hepatic lobe was significantly higher on ACSA DWI than on non-ACSA DWI (Table 2 and Table 3, Figure 2 and Figure 3). This significant SI increase was observed consistently across both respiratory conditions and in both hepatic regions. ADC values of the left lateral segment and the right hepatic lobe were significantly lower on ACSA DWI than on non-ACSA DWI in both FB and BH imaging (Table 4). For NEX 3 and 4, the SI of ACSA was significantly higher than that of non-ACSA in all regions, including the periphery and center of both the lateral segment and the right lobe of the liver (*p* < 0.0001 to 0.0297). For NEX 2, the SI of ACSA was significantly higher than that of non-ACSA in all regions except the center of the right hepatic lobe (*p* = 0.0098–0.0352). The ICC for SI measurements was 0.993 (95% confident interval [95% CI] 0.992–0.994), and that for ADC measurements was 0.972 (95% CI 0.967–0.977), both indicating excellent reliability.

The SIDR was significantly higher in the left lateral segment than in the right hepatic lobe in both FB and BH imaging (Table 5), indicating a greater relative SI increase in the left lateral segment.

The SIR and ADC ratio between the center of the right hepatic lobe and the peripheral left lateral segment were significantly smaller on ACSA DWI than on non-ACSA DWI in both FB and BH imaging (Table 6). These reductions in both SIR and ADC ratio were observed consistently across respiratory conditions.

When comparing FB-ACSA and BH-ACSA DWI, the SIR between the right hepatic lobe and left lateral segment was significantly smaller on FB-ACSA DWI (Table 7).

In contrast, no significant difference in ADC ratio was observed between FB-ACSA and BH-ACSA DWI.

## 4. Discussion

The present study demonstrates that ACSA reconstruction consistently alters liver DWI signal characteristics in both FB and BH acquisitions. Importantly, these results are derived exclusively from quantitative measurements summarized in the Tables, allowing for the direct assessment of ACSA behavior under BH conditions. A key observation is the significant increase in SI with ACSA DWI in both hepatic regions, even during BH imaging. BH DWI is commonly adopted to reduce respiratory motion; however, it inherently limits the number of excitations and remains susceptible to residual motion, particularly cardiac-induced motion affecting the left lateral segment [3,12,13,14]. The observed SI increase indicates that ACSA reconstruction enhances signal robustness even when repetition sampling is constrained, underscoring its applicability in time-limited BH protocols.

The higher SIDR observed in the left lateral segment compared with the right hepatic lobe further highlights the regional dependency of ACSA effects. The left lateral segment is known to be more vulnerable to cardiac motion and phase inconsistency [3,12,13,15], and the greater relative SI increase suggests that ACSA preferentially stabilizes the signal in motion-prone regions rather than uniformly amplifying the signal across the liver.

Another important finding is the consistently lower ADC values observed with ACSA DWI. This phenomenon has also been reported in FB abdominal ACSA studies [7,8,9] and should be interpreted in the context of noise statistics. The observed reduction in ADC values with ACSA processing likely reflects the mitigation of the Rician noise floor bias. It is well established that Rician noise leads to an artificial elevation of ADC values, particularly at high b-values where the signal is low [13,16,17]. By utilizing complex signal averaging, ACSA suppresses this noise floor, potentially yielding ADC values that are closer to the true molecular diffusion. However, as this study lacked a ground-truth phantom or histopathological correlation, these findings should be interpreted as a reduction in noise-induced bias rather than a definitive increase in absolute accuracy. The reduction in inter-lobar SIR and ADC ratios with ACSA further supports this interpretation. The liver is physiologically a relatively homogeneous parenchymal organ, and large regional SI and ADC discrepancies are more likely attributable to technical artifacts than true biological variation [18,19,20]. Therefore, the observed reduction in ADC heterogeneity suggests improved quantitative consistency rather than the distortion of diffusion information [21].

The comparison between FB and BH ACSA provides additional insight. While FB ACSA demonstrated superior signal uniformity as reflected by smaller SIR values, ADC ratios did not differ significantly between the two conditions. This indicates that ADC quantification with ACSA is robust across respiratory strategies, whereas signal uniformity benefits from the larger pool of repetitions available during FB. From a practical perspective, BH ACSA remains a viable option when patient cooperation or scan time is limited.

Regarding clinical implications, improved SI and reduced intrahepatic heterogeneity may potentially enhance the stability of liver DWI, particularly in the left lateral segment, which is a known blind spot for lesion detection [13,14]. Although lesion detectability was not assessed in this study, these findings may serve as a basis for future investigations into whether ACSA contributes to more reliable focal lesion assessment. Recent advances in the use of artificial intelligence (AI) for processing liver MRI have improved lesion detection, segmentation, and characterization, thereby supporting diagnostic and prognostic assessment. These technologies represent important complementary developments in the imaging evaluation of liver cancer. In addition, more uniform ADC measurements may enhance confidence in quantitative assessments of diffuse liver disease, fibrosis, inflammation, and treatment response monitoring [18,19,22,23]. Because ACSA is implemented purely as a post-processing technique, it can be readily integrated into routine BH liver MRI without altering acquisition protocols or increasing scan time.

This study has limitations. First, it was retrospective and single-center in design. Second, only parenchymal liver measurements were analyzed; lesion detectability under BH ACSA DWI was not directly assessed. Third, ADC behavior was evaluated at a single b-value combination, and generalizability to other diffusion settings requires further validation. Fourth, PIQE, a deep learning-based reconstruction method, was applied to both sequences and may have influenced SI, noise, and ADC in each dataset. Although deep learning-based denoising can interact with underlying signal characteristics, such effects would not preferentially benefit either reconstruction. Therefore, the observed differences are considered to arise primarily from ACSA rather than from PIQE. Finally, phantom studies and multicenter reproducibility analyses are warranted to further confirm the quantitative accuracy of ACSA-derived ADC values.

## 5. Conclusions

ACSA DWI significantly improves SI, intrahepatic uniformity, and ADC reliability even under BH liver imaging. BH ACSA DWI may represent a potentially useful application complementary to FB ACSA DWI, supporting its consideration as a post-processing strategy for improving qualitative and quantitative liver DWI in future investigations.

## Figures and Tables

**Table 1:** Demographics of study population.

Characteristics	Patients, *n* (%)
Total number of patients	62
Man/woman	35 (56)/27 (44)
Age (years) *	67.8 ± 13.6
Underlying disease	
Hepatocellular carcinoma (HCC)	16 (26)
Intraductal papillary mucinous neoplasm (IPMN)	9 (15)
Hepatic hemangioma	7 (11)
Pancreatic cancer	6 (10)
Liver metastasis	5 (8)
Cholelithiasis	5 (8)
Fatty liver	2 (3)
Normal	2 (3)
Focal nodular hyperplasia (FNH)	1 (2)
Primary sclerosing cholangitis (PSC)	1 (2)
Intrahepatic hematoma	1 (2)
Focal autoimmune pancreatitis (AIP)	1 (2)
Pancreatic pseudocyst	1 (2)
Focal pancreatic enlargement	1 (2)
Pancreatic cyst	1 (2)
Acute cholangitis	1 (2)
Choledocholithiasis	1 (2)
Gallbladder cancer	1 (2)

* Data is mean ± standard deviation.

**Table 2:** Comparison of SI of the liver between ACSA DWI and non-ACSA DWI in imaging with FB and BH.

	FB (*n* = 62)			BH (*n* = 62)		
	ACSA	Non-ACSA	*p* Value	ACSA	Non-ACSA	*p* Value
Liver, lateral segment (periphery)	22.9 (14.2, 31.9)	17.3 (12.4, 24.3)	<0.0001	51.6 (33.3, 78.9)	41.9 (26.4, 67.9)	<0.0001
Liver, lateral segment (center)	27.4 (18.3, 37.3)	22.1 (14.6, 34.7)	<0.0001	73.9 (46.8, 94.0)	60.1 (35.9, 85.0)	<0.0001
Liver, right lobe (periphery)	39.6 (27.3, 51.1)	38.5 (25.6, 51.7)	<0.0001	88.9 (73.7, 109.2)	86.4 (68.3, 108.5)	<0.0001
Liver, right lobe (center)	34.4 (23.3, 51.6)	33.1 (22.9, 51)	0.0002	91.6 (68.4, 112.8)	88.5 (65.9, 111.2)	<0.0001

Data are medians, with 25th and 75th percentile in parentheses.

**Table 3:** Comparison of SNR of the liver between ACSA DWI and non-ACSA DWI in imaging with FB and BH.

	FB (*n* = 62)			BH (*n* = 62)		
	ACSA	Non-ACSA	*p* Value	ACSA	Non-ACSA	*p* Value
Liver, lateral segment (periphery)	6.5 (4.0, 10.6)	5.1 (3.5, 7.5)	<0.0001	8.1 (5.1, 14.5)	6.7 (4.1, 11.8)	<0.0001
Liver, lateral segment (center)	7.4 (5.0, 12.4)	6.1 (4.4, 10.4)	<0.0001	10.9 (6.0, 18.3)	9.1 (5.1, 16.9)	<0.0001
Liver, right lobe (periphery)	11.5 (6.5, 16.8)	10.8 (6.2, 15.4)	0.0004	15.7 (9.1, 22.8)	14.5 (9.1, 22.2)	<0.0001
Liver, right lobe (center)	9.8 (6.3, 16.0)	9.5 (6.0, 14.2)	0.0005	14.6 (8.2, 25.5)	14.2 (8.1, 24.9)	<0.0001

Data are medians, with 25th and 75th percentile in parentheses.

**Table 4:** Comparison of ADC values of the liver between ACSA DWI and non-ACSA DWI in imaging with FB and BH.

	FB (*n* = 62)			BH (*n* = 62)		
	ACSA	Non-ACSA	*p* Value	ACSA	Non-ACSA	*p* Value
Liver, lateral segment (periphery)	1241 (1089, 1437)	1461 (1246, 1711)	<0.0001	1277 (1058, 1508)	1466 (1191, 1849)	<0.0001
Liver, lateral segment (center)	1195 (1101, 1318)	1337 (1176, 1550)	<0.0001	1177 (1018, 1356)	1251 (1101, 1609)	<0.0001
Liver, right lobe (periphery)	1121 (1037, 1185)	1130 (1056, 1228)	<0.0001	1053 (1001, 1124)	1074 (1009, 1143)	<0.0001
Liver, right lobe (center)	1038 (958, 1131)	1056 (981, 1153)	<0.0001	1022 (945, 1095)	1053 (974, 1123)	<0.0001

Data are medians, with 25th and 75th percentile in parentheses.

**Table 5:** Comparison of SIDR of the liver between the right lobe and left lateral segment.

	FB (*n* = 62)		*p* Value	BH (*n* = 62)		*p* Value
Liver, lateral segment (periphery)	17.6 (4.9, 35.1)	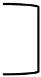	<0.0001	15.2 (4.6, 37.4)	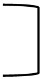	<0.0001
Liver, right lobe (periphery)	1.3 (0.2, 3.3)			1.7 (0.5, 4.2)		
Liver, lateral segment (center)	13.9 (4.4, 29.9)	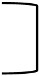	<0.0001	10.5 (4.2, 28.3)	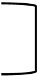	<0.0001
Liver, right lobe (center)	0.9 (−0.1, 2.8)			1.4 (0.1, 5.2)		

Data are medians, with 25th and 75th percentile in parentheses.

**Table 6:** Comparison of SIR and ADC ratio between the right lobe and left lateral segment for ACSA DWI and non-ACSA DWI in imaging with FB and BH.

	FB (*n* = 62)			BH (*n* = 62)		
	ACSA	Non-ACSA	*p* Value	ACSA	Non-ACSA	*p* Value
SIR	1.5 (1.2, 2.1)	1.9 (1.3, 2.4)	<0.0001	1.9 (1.3, 2.3)	2.2 (1.6, 2.8)	<0.0001
ADC ratio	0.82 (0.72, 0.90)	0.74 (0.60, 0.84)	<0.0001	0.81 (0.68, 0.93)	0.73 (0.61, 0.86)	<0.0001

Data are medians, with 25th and 75th percentile in parentheses.

**Table 7:** Comparison of FB and BH ACSA DWI for differences in SIR and ADC ratio between the right lobe and left lateral segment.

	FB (*n* = 62)	BH (*n* = 62)	*p* Value
SIR	1.5 (1.2, 2.1)	1.9 (1.3, 2.3)	0.0036
ADC ratio	0.82 (0.72, 0.90)	0.81 (0.68, 0.93)	0.3794

Data are medians, with 25th and 75th percentile in parentheses.

## Data Availability

The datasets generated or analyzed during the study are available from the corresponding author upon reasonable request due to ethical restrictions; access requires approval from the institutional ethics committee.

## Abbreviations

The following abbreviations are used in this manuscript:

ACSA	adaptive complex signal average
ADC	apparent diffusion coefficient
BH	breath-hold
DWI	diffusion-weighted imaging
FB	free-breathing
SI	signal intensity
SIDR	signal intensity difference ratio
SIR	signal intensity ratio
SNR	signal-to-noise ratio
